# Organic pollutants in the street dust of a European Metropolitan area

**DOI:** 10.1007/s11356-025-37355-7

**Published:** 2026-01-11

**Authors:** Miguel Velázquez-Gómez, Marcello D’Amico, Silvia Lacorte

**Affiliations:** 1 Chemometrics, Department of Environmental Chemistry, Institute of Environmental Assessment and Water Research IDAEA-CSIC, Barcelona, Spain; 2 Department of Chemical and Environmental Engineering, Underwater Vehicles Lab – LVS, Technical University of Cartagena, Cartagena, Spain; 3 Department of Conservation Biology and Global Change, Doñana Biological Station EBD-CSIC, Seville, Spain

**Keywords:** Organic pollution, Gas chromatography, Mass spectrometry, Pollutant emission, Deposition, Persistence, Spatial modelling, Road dust, Street-dust pollution

## Abstract

Street dust is a major sink for organic pollutants, yet their spatial distribution in cities remains poorly understood. This study quantified 59 organic pollutants in street dust from 19 districts across metropolitan Barcelona, Spain. We detected 41 compounds, including polycyclic aromatic hydrocarbons (PAHs), phthalates, bisphenol A (BPA), organophosphorus flame retardants (OPFRs), nicotine, and chlorpyrifos (CPS). Samples were collected via a standardized protocol and analyzed using gas chromatography coupled to tandem mass spectrometry (GC–MS/MS). We applied generalized linear models (GLMs) and used Akaike Information Criterion (AIC) to test hypotheses on anthropogenic, environmental, and management-related influences on contamination patterns. PAH concentrations were higher in areas exposed to regional winds from the airport and harbour. Local winds correlated with lower levels of phthalates, BPA, and CPS. BPA increased with population density but dropped in sun-exposed plots. PAH and OPFR levels were lower in areas cleaned with water. Nicotine was higher in plots exposed to water runoff. Results suggest pollutant persistence and redistribution, rather than emission intensity, largely shape spatial patterns. These findings highlight the need to incorporate environmental and urban-management variables into pollution assessments and offer a methodological framework applicable to other European cities facing similar environmental challenges.

**Supplementary Information:**

The online version contains supplementary material available at 10.1007/s11356-025-37355-7.

## Introduction

Street dust is a heterogeneous mixture of mineral and organic particles consisting of natural and man-made materials (Maertens et al. 2004). The composition of this heterogeneous mixture can vary among locations, mainly depending on the dominant sources of particles in each area (Fergusson and Ryan 1984; Marín Sanleandro et al. 2018). In addition, street dust is considered a significant pollution sink and therefore a major source of human exposure, especially for children, but also for adults working in urban environments (Dytłow et al. 2024). The World Health Organization (WHO) has stated that people living in urban areas suffer prolonged exposure to street-dust pollutants through dermal contact, inhalation and ingestion, with notable implications for their health (WHO 2021).

The presence of contaminants in street dust has been investigated for several decades. However, these studies have typically focused on the elemental analysis of heavy metals (Saeedi et al. 2012; Marín Sanleandro et al. 2018; Škrbić et al. 2018a, b; Hou et al. 2019), and relatively few have examined organic compounds such as polycyclic aromatic hydrocarbons (PAHs; Wang et al. 2017a, b; Amjadian et al. 2018), phthalates (Anh et al. 2019), polychlorinated biphenyls (PCBs; (Klees et al. 2015), organophosphorus flame retardants (OPFRs; (Yin et al. 2019), or pesticides (Škrbić and Marinković 2019). These organic compounds are ubiquitous pollutants, and their monitoring is recommended by international environmental and health agencies to prevent (or at least control) their emission and toxic effects on human health and the environment (IARC 2014). Most studies investigating organic pollutants in street dust have focused on the quantification of contaminant concentrations at specific survey points, often analyzing a single contaminant (e.g., endosulfan; (Balayiannis et al. 2009)) or a family of related contaminants (e.g., PCBs, phthalate esters, PAHs; (Stoyanova et al. 2013; Škrbić et al. 2016; Alves et al. 2018; Škrbić et al. 2019)). This is a highly suitable approach for research focused on specific pollutants, but it may be insufficient to provide a more general view of the contaminants potentially affecting a given area, especially when considering the complexity of the street dust sources, namely the exhaust emissions (EE) produced by vehicles and industries and non-exhaust emissions (NEE) such as “abrasion-derived particles from brakes, tyres, vehicle materials and road wear, as well as resuspended particles that were previously deposited on the road surface” (Casotti Rienda and Alves 2021).

For this reason, in the present study we chose a multi-residue extraction and analysis approach to quantify the levels of organic pollutants in street dust, including 59 contaminants belonging to six contaminant families: PAHs, plasticizers, PCBs, OPFRs, nicotine, both legacy and modern pesticides (chlorpyrifos and malathion), bisphenol A (BPA), and nicotine. All of them are common organic pollutants, widely used in the plastic manufacturing (including phthalate esters related to tyre composition), tobacco industry, and pest control sectors, respectively, and consequently they are likely to be found in street dust (Gunathilake et al. 2022) via EE and NEE anthropogenic sources, such as motorized traffic, residential heating systems or industrial activities (Saeedi et al. 2012; Škrbić et al. 2016; Wong et al. 2018). These anthropogenic sources are usually heterogeneously distributed across the landscape, potentially leading to differences in the spatial variation of contaminant concentrations, which are also indeed influenced by resuspension factors (Casotti Rienda and Alves 2021). Similarly, there are several climatological factors such as wind exposure or solar radiation (Patrón et al. 2017), and urban structure and management aspects (green areas, traffic density, street cleaning, etc.) (Cárdenas Rodríguez et al. 2016; Querol et al. 2018) connected to deposition and persistence of organic pollutants in street dust. A summary of sources and processes affecting road dust loading and emissions can be consulted on Denby et al. (2018) (Denby et al. 2018). Factors affecting the spatial variation of organic pollutants in street dust have been identified within a few works (Ouyang et al. 2013; Pang et al. 2019) and help understanding the mechanisms affecting their spatial distribution to promote efficient infrastructure management actions and thus reducing human exposure to them after risk assessment.

Focusing on pollutant emission, we hypothesize that the spatial distribution of PAHs, phthalates, BPA and OPFRs may be related to motor traffic, including vehicle erosion (Škrbić et al. 2019) or, more generally, to anthropogenic activities (Klees et al. 2015; Amjadian et al. 2018), whereas nicotine is likely to be exclusively associated to human presence (Rico et al. 2019) and CPS to green areas where pest control is commonly applied (Li et al. 2011, Jiang et al. 2016a, b). Similarly, we further hypothesize that the spatial distribution of all these pollutants may also be influenced by environmental factors affecting their deposition and/or persistence, such as wind (Irvine and Loganathan 1998; Balayiannis et al. 2009), solar radiation (Škrbić et al. 2019; Škrbić and Marinković 2019), or water runoff (Hussain et al. 2015). Finally, focusing solely on pollutant persistence, we also hypothesize that the spatial distribution of all these pollutants may additionally be influenced by management factors, such as road cleaning (Querol et al. 2018), as low humidity has shown to enhance the resuspension emissions (Casotti Rienda et al. 2023).

Most studies investigating organic pollutants in European street dust have been carried out in small cities located in Spain, France, Greece, Serbia, Germany and Lithuania, among other countries (Durand et al. 2003; Kliucininkas et al. 2011; Klees et al. 2015; Škrbić et al. 2016; Škrbić et al. 2019; Škrbić and Marinković 2019). In the present study we selected the metropolitan area of Barcelona (Spain) as a study system. This is a typical European metropolis characterized by the presence of densely populated districts, roads with high traffic volume, several green areas distributed heterogeneously, and relative proximity to major sources of pollution, such as industrial areas, an airport, a harbor and landfills. Scientific studies like the one presented here, investigating the factors affecting spatial distribution of street-dust pollutants in a European metropolitan area may provide relevant information for understanding road pollution and impacts in one of the most densely populated areas of the world.

## Materials and methods

### Chemicals and reagents

The mother solutions used as standards, extraction materials and solvents are listed in Supplementary Materials 1 (Chemicals and reagents) with their acronyms and include the 16 US-EPA’s priority PAHs, six phthalates, BPA, OPFRs, nicotine, and CPS, among others. They were prepared at 1,000, 100 and 1 µg mL^−1^ in isooctane from their commercial formulations.

### Study area, data collection and analysis

**Fig. 1:**
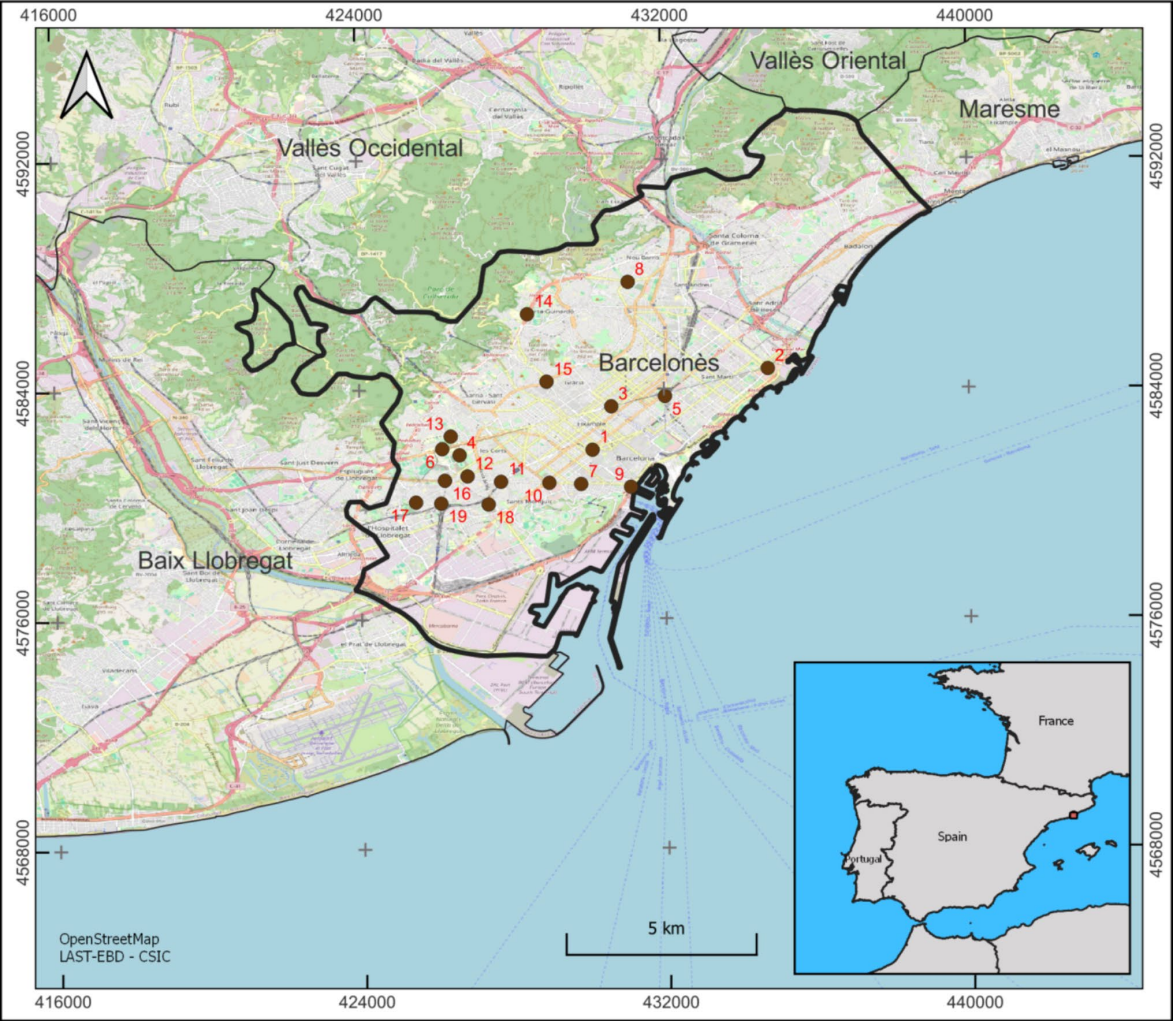
Street-dust sampling plots in Barcelona (1–14) and L’Hospitalet de Llobregat (15–19). Each sampling plot (n = 19) corresponded to a different district: 1) Antiga Esquerra de l’Eixample, 2) Diagonal Mar i el Front Marítim del Poblenou, 3) Dreta de l’Eixample, 4) Maternitat i Sant Ramón, 5) Parc i la Llacuna del Poblenou, 6) Pedralbes, 7) Poble-Sec, 8) Porta, 9) Raval, 10) Sant Antoni, 11) Sants-Montjüic, 12) Sants-Badal, 13) Sarrià, 14) Vall d’Hebron, and 15) Vila de Gràcia in Barcelona; 16) Collblanc, 17) Planes, 18) Santa Eulàlia, and 19) Torrassa in L’Hospitalet de Llobregat

The metropolitan area of Barcelona is located on the north-eastern coast of the Iberian Peninsula (41°22′57″N 2°10′37″E; Fig. 1), and the climate is typically Mediterranean, characterized by high levels of solar radiation year-round (the average annual temperature is approximately 18ºC) and most rainfall usually concentrated in autumn (the average annual precipitation is approximately 600 mm, with an average monthly peak of 96 mm in October. 1). The dominant wind is the Garbí, which blows from the south-west (Gavaldà et al. 1992, Viedma Muñoz 2001). The elevation of this metropolitan area ranges from approximately 500 m in the surrounding Collserola Mountains to sea level along the sandy coast. Barcelona is one of the largest European cities, 1,702,814 inhabitants within the city limits and 3,303,921 in the metropolitan area 2, which includes dozens of other municipalities, with L’Hospitalet de Llobregat being the largest among them (276,617 inhabitants in 2023 3). This metropolitan area encompasses districts with widely varying human-population densities, ranging from relatively low (e.g. Pedralbes, 44 inhabitants/ha) to extremely high (e.g. Torrassa, 629 inhabitants/ha). Similarly, it features districts with contrasting architectural styles, from skyscrapers to urban cottages, significant variations in traffic volumes across different roads (with several million daily trips by private motor vehicles in the metropolitan area 4), and green areas that are heterogeneously distributed. Also, industrial activities such as those developed in the port and airport are relevant as sources of pollution.

We surveyed street dust across the metropolitan area using 19 sampling plots, each in a different district (Fig. 1). The design ensured sufficient variation in the selected explanatory variables (Fig. 1). Each plot covered 100 square meters and samples were collected by using a vacuum cleaner (Bosch BKS4 14.4 V) directly on the pavement of the sampling plot. Before vacuuming, leaves, pebbles, and plastic fragments were removed. The vacuuming process lasted an average of 20 min, with some variation depending on dust abundance and the time required to reach the maximum dust collection capacity (i.e., half tank of the vacuum cleaner; (Velázquez-Gómez et al. 2018). Once collected, each sample was immediately wrapped in aluminum foil to prevent degradation, sample loss, and environmental contamination (Król et al. 2012). All samples were transported to the laboratory in a cool box to protect them from heat and humidity, which could accelerate degradation (Morawska and Salthammer 2003). After each sampling session, the vacuum cleaner was washed with a wet cloth (distilled water) and recharged to maintain optimal suction power, ensuring the reproducibility of the sampling method.

Samples were sieved through 500 µm and 125 µm sieves, preserved, and the 125 µm particle size extracted following the methodology described in previous studies (Velázquez-Gómez et al. 2018). Samples were then stored in aluminum foil-covered glass vials at room temperature until extraction and analysis. Extraction was performed using an ultrasound-assisted solid–liquid extraction process, followed by a clean-up step with Florisil. Chemical analysis was conducted using gas chromatography coupled with tandem mass spectrometry (GC–MS/MS), a method previously validated in our laboratory (Velázquez-Gómez et al. 2018) (consult supplementary information for specific data on QA/QC).

### Statistical, source analyses and health relevance

The detected concentrations (ng g^−1^) of the various organic pollutants were summarized using descriptive statistics, including the median, minima, and maxima values, as well as individual detection frequencies (%) and their standard deviations. Missing values were replaced with half of the method detection limit (MDL/2) for each compound. The concentrations of those pollutants belonging to defined chemical families were grouped into summations (PAHs, phthalates, and OPFRs), while others remained considered as individual concentrations (BPA, nicotine, CPS). Consequently, the response variables in our statistical analyses were the concentration of *PAHs*, *phthalates*, *BPA*, *OPFRs*, *nicotine*, and *CPS* (always in relation to the sampling plot).

To test our hypotheses, we established several explanatory variables related to anthropogenic activities that either promote or reduce the emission, deposition, and persistence of pollutants, namely human presence, vehicular traffic, green-area management, and road cleaning. Additionally, we considered environmental variables that specifically influence pollutant deposition and persistence, including exposure to wind, sun, and runoff water. More specifically, human presence at each sampling plot was represented by two variables at different scales: *district-population density* at the broader scale and *pedestrian-activity index* at the sampling plot scale. The former was a continuous variable reporting the official human-population density of the district where the sampling plot was located 5, while the latter was a continuous index estimating the number of people potentially walking through the sampling plot, calculated as the product of *district-population density* and *road width* at the sampling plot and then confirmed by direct observation. Vehicular traffic was represented by two alternative variables: *road width* and *traffic-volume proxy*. The former was a continuous variable measured using a geographic information system (GIS) at the sampling plot, whereas the latter was a categorical classification of *road width* based on the well-established correlation between road width and traffic volume (Jaeger et al.^2005^, D’Amico et al. 2015). Specifically, 10-m-wide roads were considered low-traffic, 15-m-wide roads were classified as medium-traffic, 20/30-m-wide roads were defined as high-traffic, and those wider than 30 m were categorized as very-high-traffic. Green-area management was represented by a continuous variable, *distance to the nearest green area*, considering that pesticides are widely used in such environments (Meftaul et al. 2020). However, the nearest green area may not necessarily be the best-connected green area to the sampling plot. Therefore, we introduced two additional explanatory variables that accounted for both the *distance to the nearest green area* and key environmental factors relevant to pollutant dispersion: topography (which influences water runoff dynamics; Hofmeister et al. 2016) and exposure to dominant winds (which blow from the south-west in our study area; Gavaldà et al. 1992, Viedma Muñoz 2001). The resulting continuous variables were *distance to the nearest upstream green area* and *distance to the nearest south-western green area*, respectively. The final explanatory variable related to anthropogenic activities was *road-cleaning management*, which may significantly affect pollutant persistence in street dust. This categorical variable had two levels, distinguishing between cleaning with and without water, based on questionnaires submitted to commercial establishments located near our sampling plots and confirmed by direct observation.

Regarding the environmental variables that either promote or reduce pollutants’ deposition and persistence, the first three were related to wind at two different scales. At a regional scale, as mentioned above, the dominant wind in Barcelona (i.e., the Garbí) blows from the south-west (Gavaldà et al.^1992^, Viedma Muñoz 2001). Consequently, based on the city topology, we used GIS to establish a categorical variable named *regional-wind exposure*, which classified sampling plots as either exposed or not to the Garbí. At a local scale, given that buildings’ height can reduce pollutant deposition (Amato et al. 2019), we defined two alternative variables related to wind exposure: *local-wind exposure* and the *local-wind-exposure index*. The former was a categorical variable with three levels (i.e., sampling plot with high exposure due to low surrounding buildings, medium exposure due to medium-height surrounding buildings, or low exposure due to high surrounding buildings), whereas the latter was an ordinal variable with three corresponding exposure ranks. Characterization of this variable was carried out through visualization using Google Street View 6, and then confirmed by direct observation. Another environmental factor considered was *sun exposure*, which can affect pollutants’ persistence (Huang et al. 2020). This categorical variable (i.e., sampling plot exposed or not to solar radiation) was determined using both GIS and Google Street View, and then confirmed by direct observation. Finally, the last environmental factor analyzed was *water*-*runoff exposure*, which can influence both pollutant deposition and persistence (Zhang et al. 2008; Grung et al. 2021). Like sun exposure, this categorical variable (i.e., whether the sampling plot was exposed to water runoff or not) was assessed using both GIS and Google Street View, and then confirmed by direct observation.

We used generalized linear models (GLMs) and evaluated their performance using the Akaike Information Criterion (AIC). For each model, the response variable was the pollutant concentration in the different sampling plots. We performed a model selection for each targeted pollutant: PAHs (i.e., the sum of all PAHs concentrations, hereafter ∑PAHs; see Table 1), phthalates (i.e., ∑phthalates), BPA, OPFRs (i.e., ∑OPFRs), nicotine and CPS (see a summary of model selection in Table 2 and the full model selection in Supplementary Materials 2 – AIC model selections). We always used a negative binomial error distribution with a log link function. In the first model selection, the response variable was the *PAHs concentration in the sampling plot*, and different hypotheses were tested through univariate GLMs, with the null model (without explanatory variables) serving as a baseline. The anthropogenic-activity hypothesis was divided into three sub-hypotheses: 1) the human-presence hypothesis, tested through a model including *district-population density* as the only explanatory variable; 2) the vehicular-traffic hypothesis, tested through two models, each including either *road width* or the *traffic-volume proxy* as the only explanatory variable; and 3) the road-cleaning hypothesis, tested through a model including *road-cleaning management* as the only explanatory variable. Similarly, the environmental-factors hypothesis was divided into three sub-hypotheses: 1) the wind-exposure hypothesis, tested through three models, each including either *regional-wind exposure*, *local-wind exposure*, or the *local-wind-exposure index* as the only explanatory variable; 2) the sun-exposure hypothesis, tested through a model including *sun exposure* as the only explanatory variable; and 3) the water-runoff-exposure hypothesis, tested through a model including *water-runoff exposure* as the only explanatory variable. In the second to fourth model selections, were the response variables were *phthalates*, *BPA*, and *OPFRs concentrations in the sampling plot*, the model selection process was identical to that used for PAHs, except that the human-presence hypothesis included two models, one incorporating *district-population density* and the other incorporating the *pedestrian-activity index* as the sole explanatory variable. In the fifth model selection, where the response variable was *nicotine concentration in the sampling plot*, the model selection followed the same approach as for phthalates, BPA, and OPFRs but excluded the vehicular-traffic hypothesis. Finally, in the sixth model selection, where the response variable was *CPS concentration in the sampling plot*, the model selection followed the same structure as for the previous pollutants but included only two anthropogenic-activity sub-hypotheses: 1) the green-area-management hypothesis, tested through a model including *distance to the nearest green area* as the only explanatory variable, and 2) the road-cleaning hypothesis. Additionally, we considered a combined hypothesis for CPS, integrating both anthropogenic activities and environmental factors, with two models, each including either *distance to the nearest upstream green area* or *distance to the nearest south-western green area* as the only explanatory variable. We selected the most supported model using AIC and calculated Akaike weights (wAIC) to estimate the relative support for each model (ranging from 0 to 1, with larger values indicating stronger support; Burnham and Anderson 2002). We considered all models with ΔAIC < 2 as the most plausible, but models with ΔAIC < 7 were also considered to have some limited support (Burnham et al. 2011). See a summary of model selection in Table 2 and the full model selection in Supplementary Materials 2 (AIC model selections).

**Table 1:** Summary statistics for target compounds detected in the samples analyzed. Detection frequency, median, minimum and maximum values (ng g⁻^1^), and standard deviation for each compound, considering all the sampling plots together. Sum for compound families is also provided

Pollutants	Detection frequency (%)	Median (Min–Max)	Standard deviation
Naphthalene	47	187 (77.3–1,256)	410
Acenaphthylene	53	27.3 (20.2–63.5)	16.0
Acenaphthene	63	25.2 (3.53–76.8)	22.7
Fluorene	63	42.5 (11.9–169)	41.6
Phenanthrene	100	225 (60.8–549)	151
Anthracene	95	19.4 (6.75–60.9)	14.6
Fluoranthene	100	273 (75–1,040)	254
Pyrene	100	436 (164–2,866)	595
1,2-benzanthracene	100	54.4 (16.7–227)	60.8
Chrysene	100	201 (60.0–398)	101
Benzo(b)fluoranthene	100	109 (23.3–318)	97.8
Benzo(k)fluoranthene	100	31.6 (6.99–120)	36.1
Benzo(a)pyrene	100	54.8 (17.0–200)	57.2
Indeno(1,2,3-cd)pyrene	100	52.4 (13.5–157)	43.5
Dibenz(a,h)anthracene	63	22.4 (11.0–45)	10.7
Benzo(g,h,i)perylene	100	185 (44.2–553)	130
ΣPAHs	100	2,176 (791–5,693)	1,298
DMP	84	93.3 (17.3–376)	101
DEP	53	471 (96.7–802)	291
DiBP	100	6,291 (752–17,894)	4,981
DBP	100	2,537 (171–2,536)	2,306
BBzP	100	333 (42–1,539)	416
DEHP	100	26,626 (5,378–92,498)	26,364
Σ6Phthalates	100	42,128 (7,380–108,675)	32,247
BPA	100	1,391 (131–5,527)	1,379
Σ10OPFRs	100	3,377 (1,455–9,245)	2,401
Nicotine	74	970 (165–4,644)	1,243
CPS	21	95 (22–99)	37.1

**Table 2:** Summary of model selection. Factors affecting *PAHs*, *phthalates*, *BPA*, *OPFRs*, *nicotine*, and *CPS concentration in the sampling plot*. Names for hypotheses, sub-hypotheses, and explanatory variables (i.e., models) are simplified compared to those in the main text. We considered all models with ΔAIC < 2 as the most plausible (i.e., fourth column), but models with ΔAIC < 7 were also considered to have some limited support (i.e., fifth column)

Hypothesis	Model	Tested for	Most plausible for	Plausible for
Anthropogenic activities				
- Human presence	District population	PAHs phthalates BPA OPFRs nicotine	BPA	PAHs phthalates nicotine
	Pedestrian activity	phthalates BPA OPFRs nicotine		BPA nicotine
- Vehicular traffic	Road width	PAHs phthalates BPA OPFRs nicotine		PAHs BPA nicotine
	Traffic volume	PAHs phthalates BPA OPFRs nicotine		PAHs nicotine
- Road cleaning	Road cleaning	PAHs phthalates BPA OPFRs nicotine CPS	PAHs OPFRs	phthalates BPA nicotine
- Green areas	Green area distance	CPS		
Environmental factors				
- Wind exposure	Regional winds	PAHs phthalates BPA OPFRs nicotine CPS	PAHs	phthalates BPA nicotine
	Local winds	PAHs phthalates BPA OPFRs nicotine CPS	phthalates CPS	PAHs nicotine
	Local wind index	PAHs phthalates BPA OPFRs nicotine CPS	phthalates BPA	PAHs nicotine
- Sun exposure	Sun exposure	PAHs phthalates BPA OPFRs nicotine CPS	BPA	PAHs nicotine
- Water runoff	Water runoff	PAHs phthalates BPA OPFRs nicotine CPS	nicotine	PAHs BPA OPFRs
Combinations (anthropogenic & environmental)				
- Green areas & wind	Distance SW	CPS		CPS
- Green areas & runoff	Distance upstream	CPS		CPS

Toxic equivalent factors (TEFs) were used to convert PAH concentrations into toxic equivalent of benzo[*a*]pyrene (TEQ_BaP_) to assess the human risk related to the exposure to contaminated street dust via incremental lifetime cancer risk (ILCR) using ingestion and dermal routes (consult supplementary information for specific data on risk assessment).

## Results

### Descriptive statistics

**Fig. 2:**
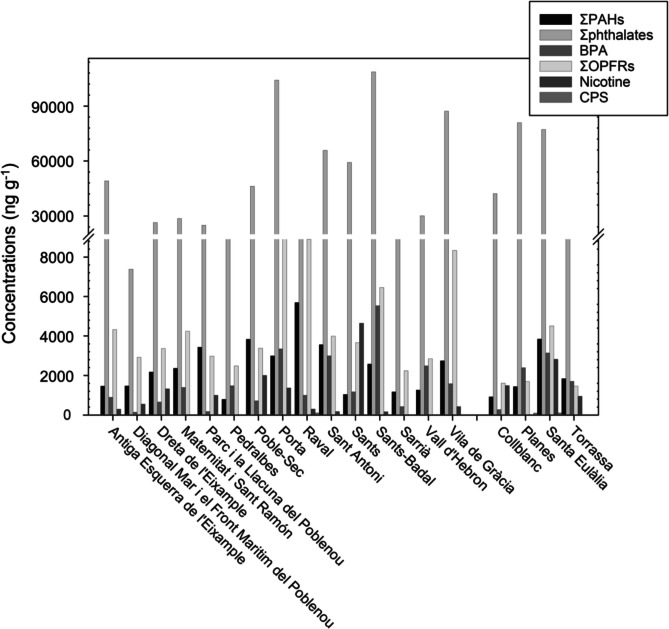
Total concentrations of each target compound or compound family detected in street dust across the 19 sampling plots in Barcelona (on the left) and L’Hospitalet de Llobregat (on the right). Each plot corresponds to a different district, and plots within each area are arranged in alphabetical order

We detected 41 out of the 59 target compounds organic pollutants in all surveyed sampling plots (Fig. 1) for the dust fraction < 125 µm (Fig. 2). Considering all plots, PAHs detection frequency ranged from 47 to 100% (Table 1, only those detected are reported), with the most abundant compounds being those with a higher molecular weight. Peak concentrations for lighter PAHs like naphthalene and phenanthrene (1,256 ng g^−1^ in Torrassa, and 549 ng g^−1^ in Raval) were also recorded, while heavy PAHs like fluoranthene, pyrene and benzo[*g,h,i*]perylene (549, 1,040, 2,866, and 553 ng g^−1^, respectively, all in Raval), and pyrene (2,866, 1,011, 980, and 763 ng g^−1^ in Raval, Sant Antoni, Poble-Sec, and Santa Eulàlia, respectively) were detected. Regarding the total (∑PAHs) in each sampling plot, it ranged from 791 ng g^−1^ in Pedralbes to 5,693 ng g^−1^ in Raval, with also high values exceeding 3,000 ng g^−1^ in Sant Antoni, Poble-Sec, Santa Eulàlia, and El Parc i la Llacuna del Poblenou. The lowest concentrations were found in Pedralbes, Collblanc, Sants, and Sarrià (791, 922, 1,040 and 1,162 ng g^−1^, respectively).

Regarding plasticizers and specifically phthalates, they were ubiquitous in all samples reaching minima and maxima total concentrations of 7,380 and 108,675 ng g^−1^ in Diagonal Mar i el Front Marítim del Poblenou and Sants-Badal, respectively (Table 1). Detection frequency was 100% for all phthalates, except for DMP and DEP, which were detected in 84% and 53% of sampling plots, respectively. DEHP and DiBP were the most abundant phthalates, with maximum concentrations of 92,498 and 17,894 ng g^−1^ in Sants-Badal and Vila de Gràcia, respectively. The lowest values were detected for DMP and BBzP (17 and 42 ng g^1^) in Sarrià and Diagonal Mar i el Front Marítim del Poblenou.

BPA had its lowest values at Diagonal Mar i el Front Marítim del Poblenou and El Parc i la Llacuna del Poblenou, at 131 and 175 ng g^−1^, respectively, and reached its maximum of 5,527 ng g^−1^ in Sants-Badal (Table 1). Considering OPFRs, the most abundant were TBOEP and TEHP, both with a 100% detection frequency (Table 1) and maximum concentrations of 4,441 and 4,206 ng g^−1^ in Vila de Gràcia and Raval, respectively. TPhP and EHDPhP were also found at relatively high concentrations, with a 100% of detection frequency. The least detected OPFRs were TCEP, TDCPP and the three TCPs although their detection frequencies were close to 90%, except for TDCPP, which had a 47% detection rate. The lowest concentrations were reached in Sants-Badal for TCEP (12 ng g^−1^), in Antiga Esquerra de l’Eixample for TCPs (approximately 25 ng g^−1^), and in Diagonal Mar i el Front Marítim del Poblenou for EHDPhP (28 ng g^−1^). Regarding ∑OPFRs, most contaminated sampling plots were Porta (9,245 ng g^−1^), Raval (8,896 ng g^−1^), Vila de Gràcia (8,337 ng g^−1^), and Sants-Badal (6,450 ng g^−1^). The least polluted areas were Torrassa (1,455 ng g^−1^), Collblanc (1,601 ng g^−1^), and Planes (1,695 ng g^−1^).

Nicotine was detected in 74% of samples, with concentrations ranging from 165 ng g^−1^ in Sants-Badal to 44,644 ng g^−1^ in Sants (Table 1). High concentrations of this compound were also recorded in Santa Eulàlia (2,816 ng g^−1^) and Poble-Sec (2,000 ng g^−1^). Finally, CPS was detected in 42% of samples, with levels ranging from 22 ng g⁻^1^ in Pedralbes to 98 ng g⁻^1^ in Raval and Santa Eulàlia.

### Model selection

**Fig. 3:**
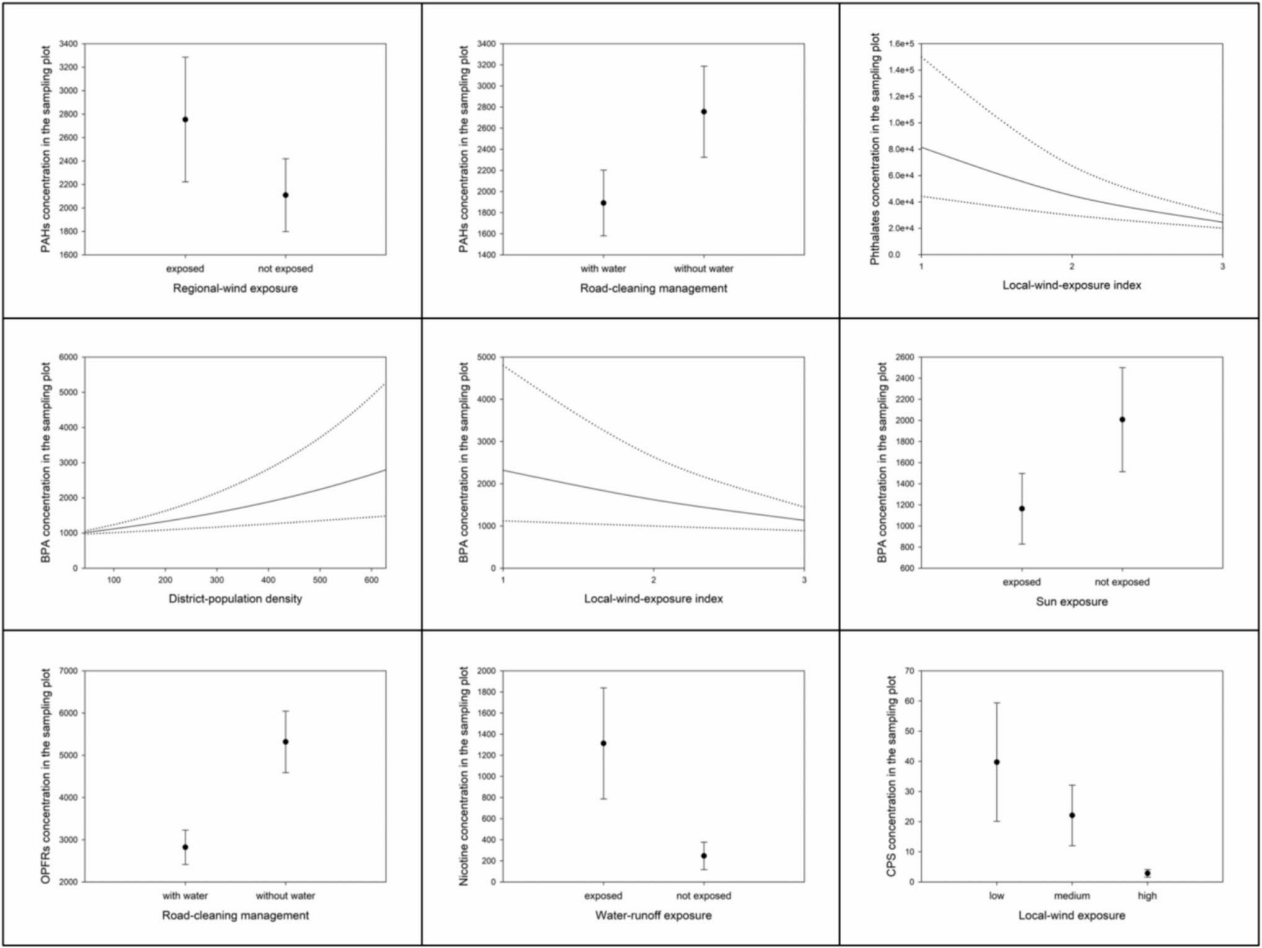
Model estimates of compound or compound family concentrations at the sampling plot, in relation to the spatial explanatory variables identified as most plausible in their respective AIC model selections

Concerning PAHs, all the implemented models had relatively high plausibility. Apart from the null model, the best supported models were the road-cleaning hypothesis, and one wind-exposure hypothesis model including *regional-wind exposure* (Supplementary Materials 2 – AIC model selections). All of them had ΔAIC < 2, indicating they had the same level of support. Consequently, both *road-cleaning management* and *regional-wind exposure* had some effect on the spatial distribution of PAHs, but since they had the same support as the null model, they were not major drivers of these pollutants. PAHs concentrations were higher in sampling plots exposed to dominant winds and cleaned without water (Fig. 3). The other plausible models, with less support (ΔAIC < 7), were the human-presence hypothesis, the vehicular-traffic hypothesis (both models), the other two wind-exposure hypothesis models, the sun-exposure hypothesis, and water-runoff exposure hypothesis.

Similarly to PAHs, almost all the implemented models for phthalates had relatively high plausibility (Supplementary Materials 2 – AIC model selections). The best supported models belonged in the wind-exposure hypothesis, and, more concretely, they were related to local-wind exposure. In fact, phthalate concentrations were considerably lower in sampling plots exposed to local winds (considering either *local-wind exposure* or the *local wind-exposure index*; Fig. 3). The other plausible models, with less support, were the human-presence hypothesis (both models), the vehicular-traffic hypothesis (only the model including *road width*), the road-cleaning hypothesis, the other wind-exposure model (including *regional-wind exposure*), the sun-exposure hypothesis, and water-runoff-exposure hypothesis.

The case of BPA was similar to the previously reported contaminants, with relatively high plausibility for all the implemented models (Supplementary Materials 2 – AIC model selections). In this case, apart from the null model, three best supported models had ΔAIC < 2: the model including *district-population density* within the human-presence hypothesis, the model including *local-wind-exposure index* within the wind-exposure hypothesis, and the sun-exposure hypothesis. As mentioned above for PAHs, the inclusion of the null hypothesis among the best supported models suggested that these factors had some effect on BPA spatial distribution, but they were not major drivers of this pollutant. However, our models showed that BPA concentrations were higher in sampling plots located in densely populated districts that were not exposed to local winds or solar radiation (Fig. 3). The other plausible models, with less support, were the other human-presence model (including the *pedestrian-activity index*, which could almost be considered one of the best supported models), the vehicular-traffic hypothesis (both models), the road-cleaning hypothesis, the other two wind-exposure hypothesis models, and water-runoff-exposure hypothesis.

Concerning OPFRs, only two of the implemented models had some plausibility, and, in fact, there was only one best supported model: the road-cleaning hypothesis (Supplementary Materials 2 – AIC model selections). Specifically, OPFRs concentrations were lower in sampling plots cleaned with water (Fig. 3). The only other plausible model, with less support, was the water-runoff-exposure hypothesis.

Regarding nicotine, all the implemented models had relatively high plausibility (Supplementary Materials 2 – AIC model selections). Apart from the null hypothesis, the best supported model was the water-runoff-exposure hypothesis. Specifically, nicotine concentrations were higher in sampling plots exposed to water runoff (Fig. 3). However, as observed for some other pollutants, the inclusion of the null hypothesis among the best supported models suggested that water runoff had some effect on nicotine spatial distribution, but it was not a major driver of this pollutant. The other plausible models, with less support, were the human-presence hypothesis (both models), the vehicular-traffic hypothesis (both models), the road-cleaning hypothesis, the wind-exposure hypothesis (all models), and the sun-exposure hypothesis.

Finally, only a few implemented models for CPS had relatively high plausibility (Supplementary Materials 2 – AIC model selections). The best supported model belonged to the wind-exposure hypothesis, and, more specifically, was the model including *local-wind exposure*. CPS concentrations were lower in sampling plots exposed to local winds (Fig. 3). The other plausible models, with less support (ΔAIC < 7), were two models combining the green-area-management hypothesis with environmental factors: one including the *distance to the nearest upstream green area* and the other including the *distance to the nearest south-western green area*.

### Health relevance

ILCR values for minima (Forum), median (Florida) and maxima (Dalt) values of TEQ_BaP_ are shown in Table SM3 2. Cancer risk was higher via dermal contact than ingestion, and overall greater for adults older than 16 years-old, although in the high polluted scenarios such as Dalt (but also Glòries, Gràcia, Paral·lel and Cruz Roja) showed a risk above the threshold of 10^–6^ (1 case of cancer per million of inhabitants) fixed by the WHO and other international health organizations (Cotruvo 1988; Maertens et al. 2008). This agrees other recent works in South European countries such as Portugal, where PAHs have been reported to reach similar concentrations in the street dust (Casotti Rienda and Alves 2021).

## Discussion

In this study, we investigated the factors affecting the spatial distribution of street-dust organic pollutants in a typical European metropolitan area: the city of Barcelona. To the author’s knowledge, some of these organic contaminants have been rarely studied, such as nicotine and CPS or have been here detected for the first time in street dust, such as BPA. Overall, our results suggested that factors related to deposition and persistence of organic pollutants (such as exposure to local winds or road-cleaning management) were more important than factors related to their emission (such as human-population density or traffic volume) in determining their concentrations in street dust. While these results do not diminish the importance of regulating the main emission sources of contaminants in typical European cities, they also shed light on additional processes influencing their concentrations in urban environments. Regarding PAHs, all the samples collected for this study can be classified as urban or suburban, which explains their levels being similar to those found in other South European cities such as Porto, Braga and Aveiro (Portugal) (Alves et al. 2018; Casotti Rienda, Nunes et al. 2023a, b) and worldwide, such as Novi Sad in Serbia (Škrbić et al. 2019), Erbil in Kurdistan (Amjadian et al. 2018), or Buenos Aires in Argentina (Cappelletti et al. 2019). Their values were below those found in megacities and areas with intense industrial activity, such as Guangzhou and Xuzhou in China (Wang et al. 2011; Wang et al. 2017a, b), Northern Vietnam (Anh et al. 2019), Guwahati in India (Hussain et al. 2015) and Rio de Janeiro and Niterói in Brazil (Franco et al. 2017). In the present work, it could be suggested that not only the traffic volume but also its fluidity condition higher emissions of PAHs, being those areas with high ∑PAHs (Dalt, Glòries, Santa Eulàlia, Paral·lel) and more traffic lights, zebra crossings and traffic jams those with more braking and therefore less efficiency of the combustion engines, as previously reported (Alves et al. 2018). PAHs partitioning among the different particle-size dust fractions has shown that the highest PAHs concentrations occur in fractions between 0.45 and 150 µm (Franco et al. 2017), (Herngren et al. 2010). Therefore, the values reported in this study (125 µm) can be considered as descriptive in terms of representativeness in each sampling plot and comparable to those from literature, being them generally in the range from 45 to 200 µm (Casotti Rienda and Alves 2021). Among the extraction methods, ultrasound assisted extraction (UAE), Soxhlet and accelerated solvent extraction (ASE) were the most commonly employed. Besides, the Mediterranean weather conditions are dry and precipitations can take place after long periods, thus favoring “the accumulation and consequent resuspension of road dust” as a common pattern in these study areas (Cunha-Lopes et al. 2022).

It is well known that atmospheric deposition is an important source of PAHs in urban areas (Škrbić et al. 2019), and in this study it was confirmed by highlighting the relative importance of exposure to the main regional wind: the Garbí. This wind blows from the south-west in Barcelona (Gavaldà et al. 1992, Viedma Muñoz 2001), potentially transporting PAHs released by both the Josep Tarradellas Barcelona–Airport and the Port of Barcelona, as well as the road vehicle exhaust resulting from their activity (Rivas et al. 2020). PAHs have already been monitored in similar locations where the combined emissions of jet fuel, heavy fuel oil for vessels and diesel oil for road vehicles coexist, such as airports and ports (Wang et al. 2022; Rodríguez-Maroto et al. 2024), and their long-range dispersion by wind has been documented (Keyte et al. 2013). Another relevant factor was road-cleaning management, with water-based cleaning reducing PAHs persistence in the sampling plot, confirming the effectiveness of this practice in limiting human exposure to some contaminants (AIRUSE 2013). The decrease in PAHs may imply that after water washing these compounds are being transferred to sewage, ultimately entering the wastewater treatment plants (Nickel et al. 2021). This fact agrees with the fact that water washing has shown high efficiency in reducing PM10 load by > 90% in a very similar sampling plot in Barcelona (Amato et al. 2009). Nevertheless, despite being consistent with current knowledge on PAH sources and transport, the limited explanatory power of our models suggests that additional, unmeasured factors may influence their spatial distribution.

All six phthalates were detected in each sampling plot, and the high concentrations of DEHP dominated the total sum of phthalates, fact already observed in PM10 from Portuguese street urban dust but at concentrations 10 times lower (Alves et al. 2018). Other study reported higher concentrations in PM10 from street dust collected in Aveiro (Portugal) using the same methodology (a resuspension chamber) (Casotti Rienda, Nunes et al. 2023a, b). In the present study, we found lower phthalates concentrations in low-density residential areas (i.e., Sarrià and Pedralbes) and close to the beach (i.e., Diagonal Mar i el Front Marítim del Poblenou), whereas the sixteen remaining sampling plots, mostly situated in high-density residential and industrial areas, showed higher concentrations. These descriptive trends were partially confirmed by our statistical analyses, aligning with the available scientific literature, which reports a decline in phthalates concentrations from urban and industrial areas to suburban and rural environments (Anh et al. 2019). However, as with PAHs, our statistical analyses confirmed that the main factor determining phthalates concentrations was more related to deposition/persistence than to emission. More specifically, phthalates concentrations were lower in the sampling plots more exposed to local winds, indicating their ability to mobilize and likely redistribute street dust containing these contaminants (Wang et al. 2017a, b). The size fraction and extraction method chosen for the analysis in the literature consulted for this discussion also corresponded to < 100 µm and UAE, ensuring comparability with our dataset.

To the best of our knowledge, BPA has been identified for the first time in street dust at median concentrations of 1,391 ng g^−1^ that indicate its common occurrence. Previous studies detected tetrabromobisphenol A (TBBPA) in dust from Chongqing (China), reporting a concentration of 74 ng g⁻^1^ (Lu et al. 2018), which is far below the levels reported here. Hence, the only available references for BPA concentrations come from soils and sediments, but it must kept in mind that indoor dust’s capacity to act as a sink for organic pollutants leads to much higher values (Sánchez-Piñero et al. 2020). BPA reached a maximum level of 90 ng g⁻^1^ in soils from public open spaces in England used for recreational purposes, which were previously utilized as landfill sites or for industrial manufacturing (Sánchez-Piñero et al. 2020). BPA was also detected in sediments from the Adyar and Cooum rivers in India, as well as the Dongyiang and Zhujiang rivers in China (Catenza et al. 2021) and Tisza and Danube rivers in Serbia (Škrbić et al. 2018a, b). Other works have focused on phenolic compounds which can have its origin in BPA, among others, and therefore may be considered good tracers for its occurrence and degradation (Alves et al. 2018).

As the main source of BPA is epoxy resins and rigid plastics such as polycarbonate, and the widespread PVC can also contain it as an additive (López-Vázquez et al. 2024), human-population density is likely a good proxy for the presence of these materials in the environment, with their degradation and wear potentially playing a major role in determining their concentrations in street dust, as described by our findings. Additionally, the other factors identified as relevant in determining BPA concentration in the sampling plot were exposure to local winds and solar radiation, both of which contributed to a decrease in pollutant persistence. As discussed earlier, local winds can mobilize and likely redistribute street dust containing phthalates; however, we could not identify anything similar regarding BPA in the available scientific literature. Nevertheless, it appears plausible that the same process may also influence this contaminant. Regarding sun exposure, the photodegradation of BPA polycarbonate has been well-established as a relevant degradation pathway for these molecules (Diepens and Gijsman 2007), and therefore, our results are broadly consistent with these experimental observations. As discussed above for PAHs, although the identified drivers for BPA are consistent with existing knowledge, the limited explanatory power of the models suggests that additional, unmeasured factors likely contribute to its spatial distribution.

OPFRs levels in the present study were higher than PAHs and lower than phthalates concentrations. This pattern aligns with the limited literature on street dust, which has been primarily reported in Asian countries. For example, OPFRs were detected at lower concentrations in some smaller cities in China (Pang et al. 2019) compared to the levels detected in our study, while similar values to ours have been recorded in Suzhou (Li et al. 2017) and Dalian (Zhang et al. 2020), both in China as well and using the same particle size (100 µm) and extraction method (UAE). Much higher values (up to one order of magnitude greater) have been detected in outdoor settled dust from a multi-waste recycling area in Tianjin, China (Wang et al. 2018), as well as in several industrial areas in Pakistan, where OPFRs pollution is significantly higher compared to rural areas (Khan et al. 2016). In our case study, the model selection once again underscored the importance of pollutant persistence over its emission. Specifically, road-cleaning management employing water-based methods reduced the persistence of OPFRs in the sampling plot, similar to the observed effect for PAHs, thereby further confirming the effectiveness of this practice in mitigating human exposure to specific contaminants (AIRUSE 2013).

Nicotine is a widespread contaminant in urban environments, with cigarette filters being the most commonly encountered item in these settings, comprising 22–46% of total visible litter (Roder Green et al. 2014). The nicotine in cigarette filters has been reported to contribute to the composition of street dust, accounting for up to 1% (w/w) of the sample weight (Hiki et al. 2017). Additionally, nicotine from third-hand smoke adhered to urban-furniture dust can reach concentrations that are equal to, or even exceed, those reported in indoor environments (Santos e Silva et al. 2016). More notably, cigarette filters release nicotine under humid conditions, with concentrations reaching 3.8 mg g⁻^1^ (Roder Green et al. 2014). Since nicotine is a polar compound that, once leached from cigarette filters, can reach aquatic environments, it has been studied in street dust as a key source of pollution in road runoff and sediments. In Japan, its concentrations in street dust were found to be of the same order of magnitude as those detected in the present study (Hiki et al. 2017). The existing literature is consistent with our findings, as we observed elevated nicotine concentrations in sampling plots exposed to water runoff. Similarly to PAHs and BPA, the spatial distribution of nicotine appears to be influenced by additional factors not captured by the selected predictors.

CPS, a widely used organochlorine pesticide and a potential persistent organic pollutant (Watts 2012), has been extensively studied in recent decades to assess its levels in indoor dust and the associated human exposure, particularly in agricultural areas where it is frequently applied (Butte and Heinzow 2002; Quirós-Alcalá et al. 2011; Dalvie et al. 2014). In fact, elevated CPS concentrations have been reported in a study from Hualien County, Taiwan (Hung et al. 2018), although they are not directly comparable to the levels found in the present study, likely due to the lower usage of this pesticide in Barcelona and other European cities. In contrast, similar detection frequencies and concentrations to those observed in this study have been reported in North American cities, such as Riverside, California (Jiang et al. 2016a, b), potentially due to its low solubility in water and rapid degradation, as informed by the National Center for Biotechnology Information 7. Additionally, its strong affinity for organic matter reduces its binding to soil or dust particles (Yao et al. 2008), and its elevated concentrations in indoor dust have led to a stronger focus on indoor environments in multi-pathway models and risk assessments (Buck et al. 2001; Pang et al. 2002), leading to the observed lack of studies in street dust compared to other pesticides. Overall, both hydrophobicity and dust adsorption rate appear to influence CPS persistence, as reflected in our model selection, which indicated that its concentrations were predominantly affected by exposure to local winds.

## Conclusions

Overall, in this study we observed that contamination levels in street dust due to organic pollutants in a typical European metropolitan area are consistent with expectations, showing values similar to those found in other cities worldwide (e.g., for PAHs, phthalates, OPFRs, and nicotine), but lower than those reported for megacities and industrial areas (e.g., for PAHs and OPFRs). Despite some limitations of our approach, this study may serve as an interesting model combining multi-residue analysis with GLM-AIC for investigating the factors affecting the spatial distribution of organic pollutants in street dust. Indeed, our results suggest that, in a typical European metropolitan context, factors related to deposition and persistence may play a more important role than those related to emissions in determining pollutant concentrations in street dust, likely because the last are significant but not as extreme as in megacities or heavily industrialized areas; this fact provides actionable insights for urban pollution mitigation. Besides, the spatial variables we analyzed were likely better descriptors of deposition and persistence than of emissions, and therefore future studies could place greater emphasis on improving the characterization of emission-related variables. In the case of Barcelona, this should include consideration of both the port and airport, which in this study appeared to be important potential sources of PAHs, whose concentrations may pose health concerns for humans, as theoretical risk assessment showed. More generally, the measurement of all considered factors could be improved through more detailed measurements at the sampling plots but also controlled condition experiments. This will require considerable time and resources, but exhaustive results could help us better understand and then limit human exposure to these pollutants. For now, this study represents a first step in that direction and a relevant contribution to the understanding of the factors that determine the spatial distribution of street-dust organic pollutants in a typical European metropolitan area: the city of Barcelona.

## Supplementary Information

Below is the link to the electronic supplementary material.

Supplementary file1 (DOCX 52.2 KB)

## Data Availability

The authors declare that the data supporting the findings of this study are available within the paper and its Supplementary Information file. Should any raw data files be needed in another format they are available from the corresponding author upon reasonable request.

## Footnotes

1 https://www.ajuntament.barcelona.cat.

2 Data from 2024: https://www.opendata-ajuntament.barcelona.cat and https://www.amb.cat

3 https://www.idescat.cat.

4 https://www.tmb.cat.

5 Obtained from: https://www.ajuntament.barcelona.cat and https://www.l-h.cat.

6 https://www.google.com/streetview.

7 https://pubchem.ncbi.nlm.nih.gov.
