# Supplementary material for: Organic pollutants in the street dust of a European Metropolitan area
Source: Environ Sci Pollut Res Int. 2026 Jan 11;33(2):591–606. doi: 10.1007/s11356-025-37355-7 (PMC12882861; doi:10.1007/s11356-025-37355-7)
Supplement: Supplementary file 1 — Supplementary file1 (DOCX 52.2 KB) [file 11356_2025_37355_MOESM1_ESM.docx]

**Supplementary Materials**

*Environmental Science and Pollution Research*

**Organic pollutants in the street dust of a European metropolitan area**

M. Velázquez-Gómez^1,2^ *†, M. D’Amico^1,3^ †, S. Lacorte^1^

^1^ Chemometrics, Department of Environmental Chemistry, Institute of Environmental Assessment and Water Research IDAEA-CSIC, Barcelona (Spain)

^2^ Present address: Department of Chemical and Environmental Engineering, Underwater Vehicles Lab – LVS, Technical University of Cartagena, Cartagena (Spain)

^3^ Present address: Department of Conservation Biology and Global Change, Doñana Biological Station EBD-CSIC, Sevilla (Spain)

* Corresponding author:

Mail address: miguel.velazquez@ieo.csic.es

† Equal contribution

**Index**

**Supplementary Materials 1 – Chemicals and reagents**

**Supplementary Materials 2 – AIC Model Selection**

**Supplementary Materials 3 – Risk assessment for PAHs in street dust**

**Supplementary Materials 4 – Quality assurance/quality control (QA/QC)**

**Supplementary Materials 1 – Chemicals and reagents**

**Target analysis of organic pollutants (extracted from (Velázquez-Gómez, Hurtado-Fernández et al. 2018))**

The following compounds were purchased from Sigma-Aldrich (Darmstadt, Germany and St. Louis, MO, USA): hexachlorobenzene (HCB, 1000 ng µL-1 in acetone), hexachlorobutadiene (HCBD, 5000 ng µL-1 in methanol), nicotine (99.1% purity), dimethyl phthalate (DMP, 99.6%), diethyl phthalate (DEP, 99.5%), 4-octylphenol (OP, solid, 99.0% purity), tris (2-chloroethyl) phosphate (TDCEP, 97.0%), diisobuthyl phthalate (DiBP, 99.0%), dibuthyl phthalate (DBP, 99.8%), malathion solution (100 ng µL-1 in cyclohexane), chlorfenvinphos solution (100 ng µL-1 in cyclohexane), butyl benzyl phthalate (BBzP, 98.0%), triphenyl phosphate (TPhP, solid, 99.0% purity), bis (2-ethylhexyl) phthalate (DEHP, 99.7%), tritolyl phosphate (TCP, 90.0%), tris (1,3-dichloroisopropyl) phosphate (TDCPP, 95.7%), 2-ethylhexyl diphenyl phosphate (EHDPhP, neat). Other compounds were purchased from Dr. Ehrenstorfer GmbH: chlorpyrifos (solid, 99.8% purity), endosulphan (α +β at 100 ng µL-1 in cyclohexane), α-hexachlorocyclohexane (α-HCH, 10 ng µL-1 in cyclohexane), β-hexachlorocyclohexane (β-HCH, 10 ng µL-1 in cyclohexane), γ-hexachlorocyclohexane (γ-HCH, 100 ng µL-1 in cyclohexane), δ-hexachlorocyclohexane (δ–HCH, 10 ng µL-1 in cyclohexane), 4-nonylphenol (NP, solid, 99.5% purity), PCB mix 3 (containing PCB28, PCB52, PCB101, PCB118, PCB138, PCB153 and PCB180 at 10 ng µL-1 in isooctan), pesticide mix 164 (containing 2,4'-DDD, 4,4'-DDD, 2,4'-DDE, 4,4'-DDE, 2,4'-DDT and 4,4'-DDT at 10 ng µL-1 in hexane), bisphenol A (BPA, solid, 98.5% purity), tris (2-butoxyethyl) phosphate (TBOEP, 95.8%), tris (2-ethylhexyl) phosphate (TEHP, 98.5%). PAH solution mix at 200 ng µL-1 in dimethyl chloride:methanol was purchased from AccuStandard, Inc. (New Haven, CT, USA). All the standards used in this study presented a purity grade higher than 90%. Acetone (Acet), n-hexane (Hex) and isooctane were acquired from Merck (Darmstadt, Germany). Isotope labeled PAHs mix, contaning naphthalene d-8, acenaphthene d-10, phenanthrene d-10, chrysene d-12 and perylene d-12 was used as internal standar (IS) and it was purchased from Sigma-Aldrich (Darmstadt, Germany and St. Louis, MO, USA). Solution mixes of the 59 selected analytes were prepared at 1000, 100, 10 and 1 ng µL-1 as well as a 10 ng µL-1 solution of the IS, all of them in isooctane.

The compounds studied belonged to different families, detailed below:

PAHs: Naphthalene (Nap), Acenaphthylene (Ace), Acenaphthene (Acp), Fluorene (Flu), Phenanthrene (Phe), Anthracene (Ant), Fluoranthene (Flt), Pyrene (Pyr), 1,2-benzanthracene (Bzt), Chrysene (Chr), Benzo(b)fluoranthene (BbF), Benzo(k)fluoranthene (BkF), Benzo(a)pyrene (BaP), Indeno(1,2,3-cd)pyrene (IcdP), Dibenz(a,h)anthracene (DahA) and Benzo(g,h,i)perylene (BghiP).

Deuterated PAHs: Isotope labeled PAHs mix, containing naphthalene d-8, acenaphthene d-10, phenanthrene d-10, chrysene d-12 and perylene d-12, was used as internal standard (IS) and it was purchased from Sigma-Aldrich (Darmstadt, Germany and St. Louis, MO, USA).

Plasticisers: dimethyl phthalate (DMP), diethyl phthalate (DEP), diisobutyl phthalate (DiBP), dibutyl phthalate (DBP), butyl benzyl phthalate (BBzP), diethyl hexyl phthalate (DEHP), octyl phenol (OP), nonyl phenol (NP) and bisphenol A (BPA).

Pesticides: hexachlorobenzene (HCB), hexachlorobutadiene (HCBD), α-hexachlorocyclohexane (α –HCH), β-hexachlorocyclohexane (β-HCH), γ-hexachlorocyclohexane (γ-HCH or lindane), δ-hexachlorocyclohexane (δ-HCH), malathion, chlorpyrifos (chlorp), chlorphenvinfos (chlorf), α-endosulfan, β-endosulfan, 2,4’-dichlorodiphenyldichloroethylene (2,4'-DDE), 4,4’-dichlorodiphenyldichloroethylene (4,4'-DDE), 2,4’-dichlorodiphenyldichloroethane (2,4'-DDD), 2,4’- dichlorodiphenyltrichloroethane (2,4'-DDT), 4,4’-dichlorodiphenyldichloroethane (4,4'-DDD), 4,4’- dichlorodiphenyltrichloroethane (4,4'-DDT).

OPFRs: tris (2-chloroethyl) phosphate (TCEP), : tris (1.3-dichloroisopropyl) phosphate (TDCPP), triphenyl phosphate (TPhP), tris (2-butoxyethyl) phosphate (TBOEP), 2-ethylhexyldiphenyl phosphate (EHDPhP), tris (2-ethylhexyl) phosphate (TEHP), tritrolyl phosphate (o,o,o-TCP, o,m,p-TCP, p,p,p-TCP).

PCBs: PCB 28, PCB 52, PCB 101, PCB 118, PCB 153, PCB 138, PCB 180.

Alkaloids: Nicotine.

**Supplementary Materials 2 – AIC Model Selection**

**Table SM2 1: 1^st^ Model Selection (ΣPAHs).** Factors affecting *PAHs concentration in the sampling plot*. Names for hypotheses, sub-hypotheses, and explanatory variables (i.e., models) are simplified compared to the main text. ΔAIC is the relative difference of a given AIC value compared to the smallest AIC value. The best supported models (ΔAIC < 2) are highlighted in darker grey, other plausible models (ΔAIC < 7) are highlighted in lighter grey. AIC weights indicate the relative support for every model (the weights of all the models in the candidate set have the sum of 1). Evidence ratio (ER) is the ratio of wAIC, comparing the best-supported model with every competing one.

| **Hypothesis** | **Model** | **AIC** | **ΔAIC** | **wAIC** | **ER** | **Rank** |
| --- | --- | --- | --- | --- | --- | --- |
| **1) Null** | Intercept | 325.42 | 0.00 | 0.26 | 1.00 | 1 |
| **2) Anthropogenic activities** |  |  |  |  |  |  |
| - Human presence | District population | 327.93 | 2.51 | 0.08 | 3.11 | 4 |
| - Vehicular traffic | Road  width | 328.19 | 2.77 | 0.07 | 3.54 | 5 |
|  | Traffic volume | 329.03 | 3.61 | 0.04 | 5.39 | 9 |
| - Road cleaning | Road  cleaning | 325.72 | 0.30 | 0.23 | 1.03 | 2 |
| **3) Environmental factors** |  |  |  |  |  |  |
| - Wind | Regional winds | 327.08 | 1.66 | 0.12 | 2.03 | 3 |
|  | Local  winds | 330.87 | 5.45 | 0.02 | 13.53 | 10 |
|  | Local  wind index | 328.24 | 2.82 | 0.06 | 3.63 | 6 |
| - Sun | Sun  exposure | 328.27 | 2.85 | 0.06 | 3.69 | 8 |
| - Water runoff | Water  runoff | 328.26 | 2.84 | 0.06 | 3.67 | 7 |

**Table SM2 2: 2^nd^ Model Selection (Σphthalates).** Factors affecting *phthalates concentration in the sampling plot*. Names for hypotheses, sub-hypotheses, and explanatory variables (i.e., models) are simplified compared to the main text. ΔAIC is the relative difference of a given AIC value compared to the smallest AIC value. The best supported models (ΔAIC < 2) are highlighted in darker grey, other plausible models (ΔAIC < 7) are highlighted in lighter grey. AIC weights indicate the relative support for every model (the weights of all the models in the candidate set have the sum of 1). Evidence ratio (ER) is the ratio of wAIC, comparing the best-supported model with every competing one.

| **Hypothesis** | **Model** | **AIC** | **ΔAIC** | **wAIC** | **ER** | **Rank** |
| --- | --- | --- | --- | --- | --- | --- |
| **1) Null** | Intercept | 447.46 | 4.42 | 0.04 | 9.12 | - |
| **2) Anthropogenic activities** |  |  |  |  |  |  |
| - Human presence | District population | 445.2 | 2.16 | 0.11 | 2.94 | 3 |
|  | Pedestrian  activity | 449.82 | 6.78 | 0.01 | 29.67 | - |
| - Vehicular traffic | Road  width | 447.33 | 4.29 | 0.04 | 8.54 | - |
|  | Traffic volume | 451.42 | 8.38 | 0.01 | 66.02 | - |
| - Road cleaning | Road  cleaning | 446.26 | 3.22 | 0.07 | 5.00 | 5 |
| **3) Environmental factors** |  |  |  |  |  |  |
| - Wind | Regional winds | 446.23 | 3.19 | 0.07 | 4.93 | 4 |
|  | Local  winds | 443.45 | 0.41 | 0.27 | 1.23 | 2 |
|  | Local  wind index | 443.04 | 0.00 | 0.33 | 1.00 | 1 |
| - Sun | Sun  exposure | 447.17 | 4.13 | 0.04 | 7.89 | - |
| - Water runoff | Water  runoff | 449.78 | 6.74 | 0.01 | 29.08 | - |

**Table SM2 3: 3^rd^ Model Selection (BPA).** Factors affecting *BPA concentration in the sampling plot*. Names for hypotheses, sub-hypotheses, and explanatory variables (i.e., models) are simplified compared to the main text. ΔAIC is the relative difference of a given AIC value compared to the smallest AIC value. The best supported models (ΔAIC < 2) are highlighted in darker grey, other plausible models (ΔAIC < 7) are highlighted in lighter grey. AIC weights indicate the relative support for every model (the weights of all the models in the candidate set have the sum of 1). Evidence ratio (ER) is the ratio of wAIC, comparing the best-supported model with every competing one.

| **Hypothesis** | **Model** | **AIC** | **ΔAIC** | **wAIC** | **ER** | **Rank** |
| --- | --- | --- | --- | --- | --- | --- |
| **1) Null** | Intercept | 323.20 | 0.00 | 0.20 | 1.00 | 1 |
| **2) Anthropogenic activities** |  |  |  |  |  |  |
| - Human presence | District population | 323.24 | 0.04 | 0.20 | 1.02 | 2 |
|  | Pedestrian  activity | 325.23 | 2.03 | 0.07 | 2.76 | 5 |
| - Vehicular traffic | Road  width | 325.84 | 2.64 | 0.05 | 3.74 | 9 |
|  | Traffic volume | 330.12 | 6.92 | 0.01 | 31.82 | - |
| - Road cleaning | Road  cleaning | 325.76 | 2.56 | 0.06 | 3.60 | 7 |
| **3) Environmental factors** |  |  |  |  |  |  |
| - Wind | Regional winds | 325.81 | 2.61 | 0.05 | 3.69 | 8 |
|  | Local  winds | 327.19 | 3.99 | 0.03 | 7.35 | - |
|  | Local  wind index | 323.96 | 0.76 | 0.14 | 1.46 | 3 |
| - Sun | Sun  exposure | 324.13 | 0.93 | 0.13 | 1.59 | 4 |
| - Water runoff | Water  runoff | 325.39 | 2.19 | 0.07 | 2.99 | 6 |

**Table SM2 4: 4^th^ Model Selection (ΣOPFRs).** Factors affecting *OPFRs concentration in the sampling plot*. Names for hypotheses, sub-hypotheses, and explanatory variables (i.e., models) are simplified compared to the main text. ΔAIC is the relative difference of a given AIC value compared to the smallest AIC value. The best supported models (ΔAIC < 2) are highlighted in darker grey, other plausible models (ΔAIC < 7) are highlighted in lighter grey. AIC weights indicate the relative support for every model (the weights of all the models in the candidate set have the sum of 1). Evidence ratio (ER) is the ratio of wAIC, comparing the best-supported model with every competing one.

| **Hypothesis** | **Model** | **AIC** | **ΔAIC** | **wAIC** | **ER** | **Rank** |
| --- | --- | --- | --- | --- | --- | --- |
| **1) Null** | Intercept | 347.03 | 5.20 | 0.06 | 13.46 | 2 |
| **2) Anthropogenic activities** |  |  |  |  |  |  |
| - Human presence | District population | 348.83 | 7.00 | 0.02 | 33.12 | - |
|  | Pedestrian  activity | 348.87 | 7.04 | 0.02 | 33.78 | - |
| - Vehicular traffic | Road  width | 349.46 | 7.63 | 0.02 | 45.38 | - |
|  | Traffic volume | 352.83 | 11.00 | 0.00 | 244.69 | - |
| - Road cleaning | Road  cleaning | 341.83 | 0.00 | 0.79 | 1.00 | 1 |
| **3) Environmental factors** |  |  |  |  |  |  |
| - Wind | Regional winds | 349.88 | 8.05 | 0.01 | 55.98 | - |
|  | Local  winds | 352.74 | 10.91 | 0.00 | 233.92 | - |
|  | Local  wind index | 349.87 | 8.04 | 0.01 | 55.70 | - |
| - Sun | Sun  exposure | 349.82 | 7.99 | 0.01 | 54.33 | - |
| - Water runoff | Water  runoff | 347.86 | 6.03 | 0.04 | 20.39 | 3 |

**Table SM2 5: 5^th^ Model Selection (Nicotine).** Factors affecting *nicotine concentration in the sampling plot*. Names for hypotheses, sub-hypotheses, and explanatory variables (i.e., models) are simplified compared to the main text. ΔAIC is the relative difference of a given AIC value compared to the smallest AIC value. The best supported models (ΔAIC < 2) are highlighted in darker grey, other plausible models (ΔAIC < 7) are highlighted in lighter grey. AIC weights indicate the relative support for every model (the weights of all the models in the candidate set have the sum of 1). Evidence ratio (ER) is the ratio of wAIC, comparing the best-supported model with every competing one.

| **Hypothesis** | **Model** | **AIC** | **ΔAIC** | **wAIC** | **ER** | **Rank** |
| --- | --- | --- | --- | --- | --- | --- |
| **1) Null** | Intercept | 289.94 | 1.84 | 0.15 | 2.51 | 2 |
| **2) Anthropogenic activities** |  |  |  |  |  |  |
| - Human presence | District population | 292.66 | 4.56 | 0.04 | 9.78 | 7 |
|  | Pedestrian  activity | 292.73 | 4.63 | 0.04 | 10.12 | 9 |
| - Road cleaning | Road  cleaning | 292.68 | 4.58 | 0.04 | 9.87 | 8 |
| **3) Environmental factors** |  |  |  |  |  |  |
| - Wind | Regional winds | 290.59 | 2.49 | 0.11 | 3.47 | 4 |
|  | Local  winds | 291.55 | 3.45 | 0.07 | 5.61 | 6 |
|  | Local  wind index | 290.56 | 2.46 | 0.11 | 3.42 | 3 |
| - Sun | Sun  exposure | 290.76 | 2.66 | 0.10 | 3.78 | 5 |
| - Water runoff | Water  runoff | 288.10 | 0.00 | 0.37 | 1.00 | 1 |

**Table SM2 6: 6^th^ Model Selection (CPS).** Factors affecting *CPS concentration in the sampling plot*. Names for hypotheses, sub-hypotheses, and explanatory variables (i.e., models) are simplified compared to the main text. ΔAIC is the relative difference of a given AIC value compared to the smallest AIC value. The best supported models (ΔAIC < 2) are highlighted in darker grey, other plausible models (ΔAIC < 7) are highlighted in lighter grey. AIC weights indicate the relative support for every model (the weights of all the models in the candidate set have the sum of 1). Evidence ratio (ER) is the ratio of wAIC, comparing the best-supported model with every competing one.

| **Hypothesis** | **Model** | **AIC** | **ΔAIC** | **wAIC** | **ER** | **Rank** |
| --- | --- | --- | --- | --- | --- | --- |
| **1) Null** | Intercept | 148.47 | 6.22 | 0.03 | 22.42 | 4 |
| **2) Anthropogenic activities** |  |  |  |  |  |  |
| - Green areas | Distance | 150.14 | 7.89 | 0.01 | 51.68 | - |
| - Road cleaning | Road  Cleaning | 151.09 | 8.84 | 0.01 | 83.10 | - |
| **3) Environmental factors** |  |  |  |  |  |  |
| - Wind | Regional winds | 151.31 | 9.06 | 0.01 | 92.76 | - |
|  | Local  Winds | 142.25 | 0.00 | 0.76 | 1.00 | 1 |
|  | Local  winds’ index | 150.71 | 8.46 | 0.01 | 68.72 | - |
| - Sun | Sun | 151.28 | 9.03 | 0.01 | 91.38 | - |
| - Water runoff | Water  runoff | 149.58 | 7.33 | 0.02 | 39.06 | - |
| **4) Combinations** |  |  |  |  |  |  |
| - Green areas  & Wind | Distance  SW | 146.98 | 4.73 | 0.07 | 10.64 | 2 |
| - Green areas  & Water runoff | Distance upstream | 147.03 | 4.78 | 0.07 | 10.91 | 3 |

**Supplementary Materials 3 – Risk assessment for PAHs in street dust**

**Table SM3 1:** Exposure scenario for the CR assessment (Velázquez-Gómez and Lacorte 2020)

| Age group (years) | Weight (kg) | Ingestion daily intake  (mg day^-1^) | Exposure duration (years) | Dermal exposure area (cm^2^ day^-1^) | Dermal adherence factor (mg cm^-2^) | Dermal absorption fraction (m^3^ kg^-1^) |  |
| --- | --- | --- | --- | --- | --- | --- | --- |
| [Birth.3) | 8.8 | 60 | 0.5 | 2800 | 0.2 | 0.13 |  |
| [3-5) | 14.5 | 60 | 3.5 | 2800 | 0.2 | 0.13 |  |
| [5-16) | 43 | 60 | 11 | 2800 | 0.2 | 0.13 |  |
| [16-31) | 65 | 30 | 23.5 | 5700 | 0.07 | 0.13 |  |
| [31-51) | 71.2 | 30 | 41 | 5700 | 0.07 | 0.13 |  |
| [51-81] | 70 | 30 | 66 | 5700 | 0.07 | 0.13 |  |

**Equations for health risk assessment**

Toxic equivalency factors (TEFs) and total toxic benzo[a]pyrene (BaP) equivalent concentration (TEQ) were used to characterize the cancer risk posed by PAHs using the following equations [Eq. (1)]:

$\sum{TEQ}_{BaP}=\sum C_{i}\cdot{TEF}_{i}$ (1)

Where C_i_ is the concentration of each individual PAH (ng g^-1^ dw) and total TEQ is the total toxic BaP equivalent concentration (ng g^-1^ dw) of the 16 individual PAHs which presents the toxic potency of total PAHs.

${ILCR}_{ingestion}=\frac{C\cdot\left( {CSF}_{ingestion}\cdot\sqrt[3]{\frac{BW}{70}} \right)\cdot{DI}_{ingestion}\cdot EF\cdot ED}{BW\cdot AT\cdot{10}^{9}}$ (2)

${ILCR}_{dermal}=\frac{C\cdot\left( {CSF}_{dermal}\cdot\sqrt[3]{\frac{BW}{70}} \right)\cdot SA\cdot AF\cdot ABS\cdot EF\cdot ED}{BW\cdot AT\cdot{10}^{9}}$ (3)

Being C the above calculated ${TEQ}_{BaP}$ and CSF values 7.2 and 25 Kg day mg^-1^ for ingestion and dermal exposure pathways, respectively.

**Table SM3 2. Cancer risk expressed as ILCR values for minima (Forum), median (Florida) and maxima (Dalt) concentrations of TEQ_BaP_. Bold-highlighted values represent those above the limit considered safe.**

| Forum (minimum TEQ) | Ingestion | 4,6703E-09 | 2,3434E-08 | 3,5682E-08 | 2,8937E-08 | 4,7512E-08 | 7,7354E-08 |
| --- | --- | --- | --- | --- | --- | --- | --- |
|  | Inhalation | 3,0185E-13 | 1,5146E-12 | 2,3062E-12 | 7,4812E-12 | 1,2283E-11 | 1,9998E-11 |
|  | Dermal | 1,9406E-08 | 9,7376E-08 | 1,4827E-07 | 1,7135E-07 | 2,8133E-07 | 4,5803E-07 |
|  | Cancer risk | 2,4077E-08 | 1,2081E-07 | 1,8395E-07 | 2,0029E-07 | 3,2885E-07 | 5,354E-07 |
| Florida (median TEQ) | Ingestion | 2,5924E-08 | 1,3008E-07 | 1,9806E-07 | 1,6063E-07 | 2,6373E-07 | 4,2938E-07 |
|  | Inhalation | 1,6755E-12 | 8,4074E-12 | 1,2801E-11 | 4,1527E-11 | 6,8182E-11 | 1,1101E-10 |
|  | Dermal | 1,0772E-07 | 5,4052E-07 | 8,2301E-07 | 9,5111E-07 | **1,5616E-06** | **2,5425E-06** |
|  | Cancer risk | 1,3365E-07 | 6,7061E-07 | **1,0211E-06** | **1,1118E-06** | **1,8254E-06** | **2,9719E-06** |
| Dalt (maximum TEQ) | Ingestion | 8,7834E-08 | 4,4073E-07 | 6,7106E-07 | 5,4422E-07 | 8,9354E-07 | **1,4548E-06** |
|  | Inhalation | 5,6769E-12 | 2,8485E-11 | 4,3372E-11 | 1,407E-10 | 2,3101E-10 | 3,761E-10 |
|  | Dermal | 3,6497E-07 | **1,8313E-06** | **2,7884E-06** | **3,2225E-06** | **5,2909E-06** | **8,6141E-06** |
|  | Cancer risk | 4,5281E-07 | **2,2721E-06** | **3,4595E-06** | **3,7668E-06** | **6,1846E-06** | **1,0069E-05** |

**Supplementary Materials 4 – QA/QC**

The specific conditions can be consulted in Velázquez-Gómez, Hurtado-Fernández et al. (2018). Procedural blanks were included in each batch to monitor and control background contamination, and detection and quantification limits (MDLs, MQLs) were calculated as the proportional concentrations to a signal-to-noise (SNR) equal to 3 or 10, respectively, based on the average response of the QC sample spiked with the 59 standards and 100 ng of internal standard (IS); MDLs varied from 0.19 ng g^-1^ (PCB52) to 10.3 ng g^-1^ (nicotine).

The extraction method was also validated through the evaluation of linearity, precision and a potential matrix effect. The results obtained in terms of instrumental repeatability were within the range 0.39–6.91% at 800 ng mL^−1^ and from 6.35 to 37.7% at 10 ng mL^−1^. Intermediate precision for the compounds under study was acceptable, with values lower than 11.5% and 20.6% at 800 ng mL^−1^ and 10 ng mL^−1^, respectively. The matrix effect was negligible (from −20% to 20%) for 44 of the compounds under study (75%). More information regarding quality assurance and quality control (QA/QC) measures can be consulted elsewhere (Velázquez-Gómez, Hurtado-Fernández et al. 2018).
